# Assembly of large area crack free clay porous films†

**DOI:** 10.1039/c7ra11969k

**Published:** 2018-01-03

**Authors:** Xiayun Huang, Nina Ivanova, Andrea Strzelec, Nicole S. Zacharia

**Affiliations:** State Key Laboratory of Molecular Engineering of Polymers, Department of Macromolecular Science, Fudan University Shanghai 200433 China; Department of Mechanical Engineering, Texas A&M University College Station TX 77843 USA astrzelec@tamu.edu; Department of Chemical and Materials Engineering, University of Alberta Alberta Canada; Texas A&M Transportation Institute, Texas A&M University College Station TX 77843 USA; Department of Polymer Engineering, University of Akron Akron OH 44325 USA nzacharia@uakron.edu

## Abstract

Porous materials with well-defined porosity have advantages in a wide range of applications, including filtration media, catalysis, and electrodes. The bottom-up fabrication of inverse opals have promised to provide those nanostructures, but fabrication of these materials is often plagued with large numbers of defects and macro-scale cracks. Here, we present a method for making nanostructured porous clay films with well defined pore size that are crack free over a large area (multiple cm^2^).

## Abstract 2

A method for making inverse opal-like porous clay films that are crack-free over a large area (on the scale of square centimeters).

## Graphical abstract

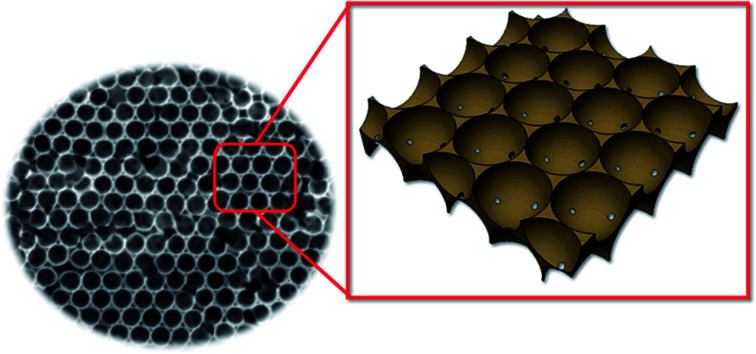

Porous media have a wide variety of applications in photonics, tissue engineering, sensing, energy devices, catalysis, filtration, and separations amongst others.^1–6^ For some applications, such as automotive particulate filters,^7–10^ well defined porosity is desired. Bottom up assembly of sub-micrometer colloidal spheres has long promised to lead to a better way to create such materials with new properties for a range of applications. One of the challenges associated with bottom up assembly, however, that has prevented this from becoming a reality, is that these types of self-assembled structures tend to have increasing amounts of uncontrolled defects as the size of the assembly increases.^11,12^ One problem associated with ordered films from colloidal particles is that as the films dry cracks form,^12^ which limits the ability to fabricate large area or very thick photonic crystals or similar materials for other applications, such as integrated optics. These cracks develop during the film formation due to the normal stress imposed by the solvent that creates a transverse tensile stress in the plane of the film that is greater than the strength of the network of the particles making up the film.^13^ Generally, for thicker films, these forces are higher, so there is often some critical thickness value above which films begin to crack upon drying.^14^ When specifically discussing the drying of films made from latex particles, one source of stress is the shrinkage of the particles themselves as the film dries, and the constraint imposed by the rigid substrate.^6^

In order to reduce and ideally eliminate crack formation during the fabrication of inverse opal porous films, a co-assembly method was first developed by Meng *et al.*^15^ in order to fabricate ordered porous colloidal crystal films *via* a mixture of colloidal sphere and ultra-small particles in one step. Using an evaporation induced assembly by particles being driven to the meniscus and then ordering, the ultra-small particles infiltrated into the voids of the colloidal crystal formed by the larger particles during the co-assembly. Other examples of binary colloidal crystals also have been reported.^16^ Going beyond the co-assembly of binary particles, studies of the cracking mechanism and development of crack free colloidal films as large as square centimeters have been reported by the co-assembly of colloidal spheres and precursors, including polymer precursors^6,17,18^ or inorganic material precursors.^1,17,19^ The spheres used to form the colloidal crystal are a sacrificial template material that is later removed, and a large area inverse opal colloidal crystal film can be achieved. Low crack inorganic films can be achieved this way, but of low film thickness.^20^ However, utilizing 2D materials or particles with high aspect ratio as the infiltrant into the voids of the colloidal crystal remains challenging, not only to fabricate large area and thick crack free films, but in ensuring that the infiltrant does not disrupt the nearly close-packed assembly of the larger spheres, retaining order and connectivity.

Here, we demonstrate a co-assembly method for making ordered inverse opal-like porous clay films that are crack free over a large area (on the scale of square centimeters). To fabricate these inverse opal-like porous films, laponite nanoplates are co-assembled into colloidal crystals with polystyrene latex particles, which are used as a sacrificial template. A critical ratio of clay to polystyrene (PS) was determined to be necessary to create crack free versions of these films. These materials have been fabricated with polystyrene opal colloidal templates either ∼500 nm or ∼1000 nm in size, but this is a generalizable method that can be made with colloidal particles of a much larger or smaller size.

Monodisperse polystyrene colloidal particles were synthesized (576 ± 7 nm and 1031 ± 15 nm) using as emulsion polymerization approach.^21^ Relative uniformity of particles size is required for their ability to pack, and to make a high quality colloidal crystal. The standard deviations of both particles are ∼3%. Using a co-assembly approach, laponite nanoplates, at the same time, were packed into the voids between polystyrene particles. Laponite is a kind of synthetic clay of trioctahedral smectitie of hectorite type, with a typical disk-shape platelet and the composition of Na_0.7_^+^[(Si_8_Mg_5.5_Li_0.4_)O_20_(OH)_4_]_0.7_^−^.^22^ An individual Laponite disk has a thickness of 0.92 nm, a diameter of about 25 nm, and a negative surface charge density of 0.014 e^−^ Å^−2^ in water.^23^ The nanoplates prefer to have lamellae stacked in a parallel orientation along the (001) plane (Fig. 1). As shown in the inset image of nanobeam diffraction (NBD) pattern, the interspacing in between the lamella layers is 3.2 Å, corresponding to the 001 reflection.^22^

**Fig. 1:**
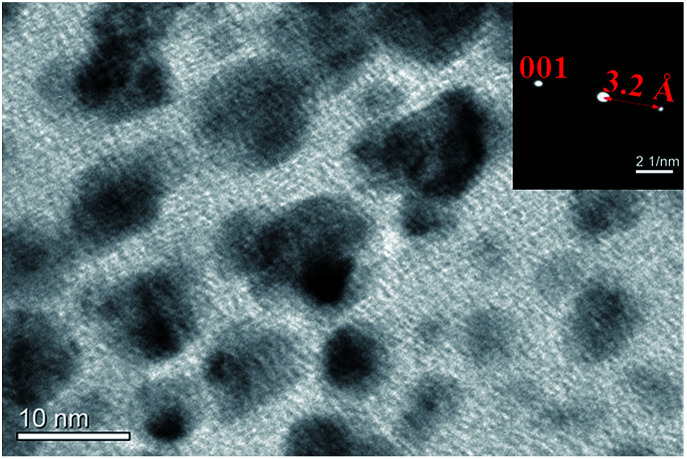
Laponite nanoplate TEM image of clay nanoplates and nanobeam diffraction pattern of a single platelet (inset).

Composite polystyrene/laponite (PS/Clay) films were assembled by convective evaporative self-assembly of mixed aqueous dispersions. Both glass and silicon wafers were used as substrate, as well as the inside of glass capillary tubes. That is to say that it is possible to co-assemble these particles on flat as well as curved substrates. Fig. 2A describes the process to create the clay inverse opal materials. A substrate is placed perpendicularly in a dispersion of laponite nanoplates and polystyrene particles. As the solution evaporates, the particles are carried to the meniscus. The larger polystyrene particles pack into a close packed configuration and the laponite fills in the interstitial voids between spheres. After the film has dried, it is heated to 500 °C in order to remove the polystyrene (Fig. S1†), leaving a porous inverse opal-type structure.

**Fig. 2:**
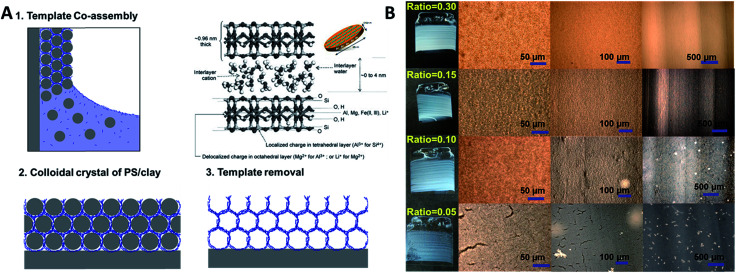
Crack free polystyrene (PS)/clay co-assembly. (A) Schematic representation of porous material fabrication. Clay platelets and PS particles in a solution are drawn to a meniscus as the solvent evaporates, creating a crystal of spherical particles with clay particles in the interstitial spacing. The PS particles are then removed by calcination, leaving behind the inverse opal structure made of clay; (B) optical micrographs of porous clay films assembled with different weight ratios of laponite to polystyrene. The sufficient amounts of laponite prevent the formation of those cracks. When the weight ratio of laponite to polystyrene is as low as 0.05, cracks are still present. The film formed with 0.01 ratio also shows some defects, but films formed with ratios of 0.15 and 0.30 are crack free over the scale of several square centimetre. The diameter of polystyrene spheres used here is ∼1000 nm. And the glass slide present here is 2.5 cm × 2.5 cm.

Changes in film quality resulted from varying the ratio of laponite to polystyrene. Films formed solely from polystyrene spheres under the assembly conditions used in this work contained macroscopic cracks,^15,24^ as did films containing only a small amount of laponite nanoplates (Fig. 2B). A sufficient amount of void filling material eliminates the formation of cracks. The macroscopic cracks are no longer observed when the weight ratio of laponite to polystyrene is above 0.15. Fig. 2B shows the calcined porous films (with the polystyrene sphere template removed) formed with a range of laponite to polystyrene ratios. It can be clearly seen that a laponite/polystyrene ratio of 0.05 creates films with cracks. A ratio of 0.1 forms films that are still not entirely defect free, but at ratios above 0.15 (such as 0.15 and 0.30 shown in Fig. 2B and 0.90 shown in Fig. 3) the films are totally crack free over the several square centimeters. The ratio of laponite to polystyrene in-between 0.15 and 0.9 leads to the open pores crack free colloidal crystal film. Here, the laponite nanoplates tend to fill in the PS interconnected voids and totally then fulfilling the voids with raising the laponite concentration. A further increase in the amount of laponite, by increasing the weight ratio of laponite to polystyrene, leads to more and more laponite nanoplates infiltrating the voids. At some point, however, there is an excess of laponite compared to the void volume. At this point, the co-assembly of laponite allows them to be stacked together (Fig. S2†) in between or at the surface of the PS spheres, and eventually the laponite wall grow thicker and thicker and the result is a closed pore material with isolated pores after removal of the polystyrene templates. The ordered porous film is fragile, however. When formed on a flexible substrate and deformed, one can see a homogenous distribution of cracks domains (Fig. S3†). This is the result of by bending ∼10 degrees on a PS substrate. Although the film is not very bendable, it is possible to form large area and thick films without cracks.

**Fig. 3:**
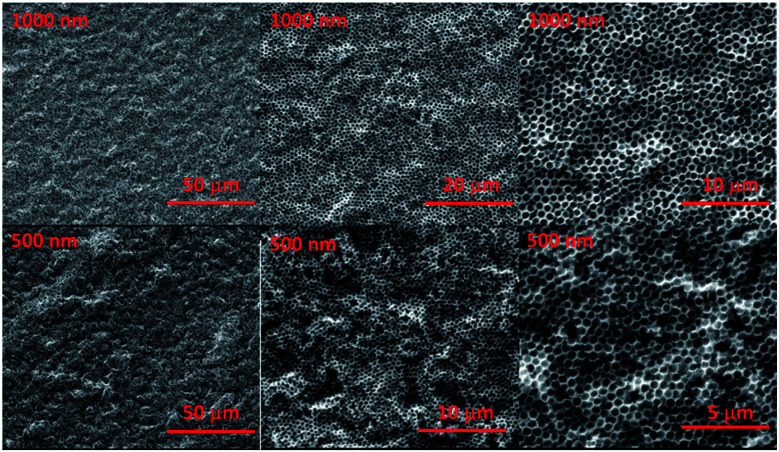
SEM images of porous film formed with ∼1000 nm PS and ∼500 nm PS. The weight ratio of laponite to polystyrene is 0.90. The images show clearly that after calcination, the porous structure is intact, having neither cracked nor collapsed.

Our results clearly indicate the possibility of introducing the multi-layered nanoplates aligned in the voids. The colloidal/nanoplate co-assembly process also offer the significant improvement of the film quality, without cracking induced *via* dehydration induced contraction and associated local capillary force during drying.^25^ As the nanoplate suspension concentrates during the drying process it undergoes a liquid–sol–gel transition with an accompanying viscosity increase.^26^ This viscous suspension then provides a glue and necking to the interconnected colloid and prohibits the formation and propagation of the cracks, regardless of colloidal size. When decreasing the size of co-assembled colloidal sphere, the similar inverted opal and crack free porous film was also fabricated by simply tuning the laponite/PS ratio (Fig. 3). At low laponite concentration, the cracks of the film were observed; while the cracks were prohibited only and high quality film with open interconnected pores was developed, when the concentration of laponite (the ratio of laponite to PS) is high enough. Further raising the laponite concentration to an extreme high value, the growing wall and non-interconnected pores were observed.

The high quality porous film not only performs the crack film and open interconnected pores, but also has a certain degree of periodicity. The selected area fast Fourier transform (FFT) of SEM image (Fig. 4A) presents the ordered hexagon pattern (insert image of Fig. 4A), meaning that the pores are ordered distributed with the same pore size and in the hexagon orientated close packing. The TEM image (Fig. 4B) of crashed aggregates showed that each pore was interconnected with each other and with the multi-layered aligned laponite walls filled inside the voids and perpendicular to the centre of the sphere. Although some periodicity is achieved, perfect single crystalline films are not fabricated, and some point defects can be seen as well. One explanation for this may be the increase in viscosity of the solution during the drying process that might “freeze” the polystyrene templates in place before they can pack more perfectly.

**Fig. 4:**
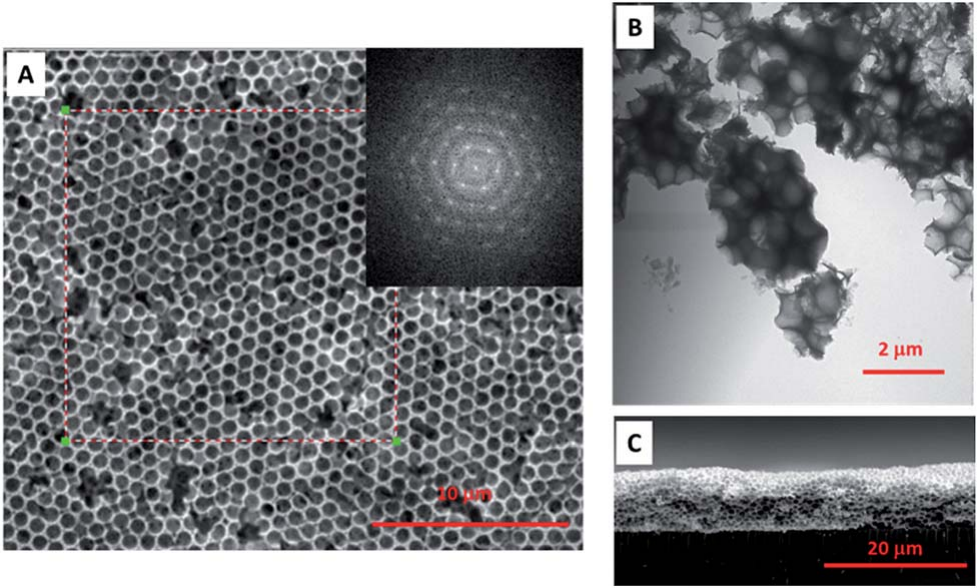
All clay porous film. (A) Top view SEM image of all clay porous film. The pores are ordered distributed and come out with hexagon FFT pattern (insert image). (B) TEM image of crashed aggregates. Each pore is interconnected with stacked laponite walls at the interface. (C) Cross-sectional view of all clay porous film, representing the crack free and pores interconnection is from the top to the bottom even the thickness is about 10 μm.

As mentioned, the crack developed due to the normal stress imposed by the solvent evaporation and interfacial tension.^13^ Likely, the crack free film was formed due to the smaller capillary stress imposed during solvent evaporation, which depends on film thickness, particle shear modulus, particle packing configuration, and the liquid–air interfacial tension.^24^ And among them, the film thickness is one of the important factors influencing the film quality. In order to avoid cracking and obtain thicker colloidal films, suspension chemistry, drying environment as well as the substrate material has been manipulated. However, for thicker films, these forces are higher, so there is often some critical thickness value above which films begin to crack upon drying.^14^ To our knowledge the threshold thickness of crack free film that begins to crack is in the range of few hundred nanometers, such as ∼500 nm reported by Aizenberg *et al.*,^1^ and ∼300 nm reported by Lee *et al.*^27^ In our system, the thickness can be easily increased to ∼10 μm and thicker. The cross-sectional view of porous film also showed the high quality crack free film perpendicularly with the pores interconnected from the top to the bottom (Fig. 4C). One might speculate that the hygroscopic nature of the laponite might slow the film drying and therefore mitigate crack formation. Another speculation is that the stacking and overlap of clay platelets distributes the stresses evenly, mitigating crack formation. Here, our thick films are created by high solution concentrations. This and other factors such as substrate tilt angle are known to control film thickness of colloidal crystals during convective self-assembly.^28,29^

In summary, we have successfully developed a co-assembly method to fabricate crack free, inverse opal-like porous clay films over a large area; on the scale of square centimeters and as thick as tens of microns. Laponite nanoplates are co-assembled with polystyrene spheres and driven into the voids of colloidal crystals, which results in crack free and inverse opal-like structured films that can be fabricated at a critical ratio of clay to polystyrene (PS). This co-assembly approach is versatile and can be utilized to crack free films with a variety of pore sizes (based on template size) and film thickness (based on overall dispersion concentration).

## Conflicts of interest

There are no conflicts of interest to report.

## Supplementary Material

RA-008-C7RA11969K-s001
