# Supplementary material for: Assembly of large area crack free clay porous films
Source: RSC Adv. 2018 Jan 3;8(2):1001–4. doi: 10.1039/c7ra11969k (PMC9076984; doi:10.1039/c7ra11969k)
Supplement: RA-008-C7RA11969K-s001 [file RA-008-C7RA11969K-s001.pdf]

Electronic Supplementary Material (ESI) for RSC Advances.
This journal is © The Royal Society of Chemistry 2017

Assembly of Large Area, Crack Free Clay Porous Films

Xiayun Huang,[a] Nina Ivanova,[b,c] Andrea Strzelec,[c,d]* and Nicole S. Zacharia[e]*

[a]*State Key Laboratory of Molecular Engineering of Polymers, Department of Macromolecular Science, Fudan University, Shanghai, China, 200433.*

[b]*Department of Mechanical Engineering, Texas A&M University, College Station, TX. 77843.*

[c]*Department of Chemical and Materials Engineering, University of Alberta, Alberta, Canada*

[d]*Texas Transportation Institute, Texas A&M University, College Station, TX, 77843.*

[e]*Department of Polymer Engineering, University of Akron, Akron, OH, 44325*

1. Experimental

Materials. Laponite RDS was obtained from Southern Clay Products. Styrene, potassium persulfate, and ethanol were obtained from Sigma Aldrich and used without further purification.

Synthesis of PS colloids. Monodispersed colloidal particles of PS (576 nm that is~500 nm; and 1031 nm that is~1000 nm; both are in mean diameter with approximately standard deviation of 3%) were synthesized by emulsion polymerization initiated by potassium persulfate.[1] The reaction was under N_2 flow and washed with ethanol several time before the PS colloids were dried.

Colloidal template co-assembly and inverse opal structure. The porous all-clay inverse opal structure was prepared based on the hard template and convective evaporation methods using PS colloids. The clay and PS colloids stock solution (1 wt%) was prepared and mixed in the various ratio and the mixture was sonicated for 30 min before the convective evaporation. Convective evaporation was performed under minimized mechanical vibration. Polystyrene sphere was removed by thermal degradation at 500 ℃ at a slow ramping time of 1 ℃/min.

Characterization. Transmission electron microscopy (TEM) images of Laponite nanoplate were taken using a JEOL JEM-2010 microscope operated at 200 kV coupled with Gatan SC 1000 ORIUS CCD camera. The dispersed clay suspension was sonicated at 4℃ for 15 min. Laponite suspension was dropped onto the 200 mesh carbon film which is supported by copper grids (TED PELLA, Inc). All TEM images were taken at the same trends of magnification modulation as these of calibration samples. Scanning electron microscopy (SEM) images were acquired using an FEI Quanta 600 field emission scanning electron microscope with an accelerating voltage of 10 kV. The cross-sectional image was performed using titling and rotational stage. Optical microscopy was performed with a Carl Zeiss optical microscope (model no. 430014-9902) equipped with an Axiocam imaging system (AXIO M2m). Thermogravimetric analysis (TGA) was performed with a Q500 TGA (TA Instruments, West Sussex, UK). Samples were dried in vacuum to remove unbound water and equilibrate the system to be ramped to 800 °C at 5 °C/min under air flow.

2. TGA of Polystyrene Spheres

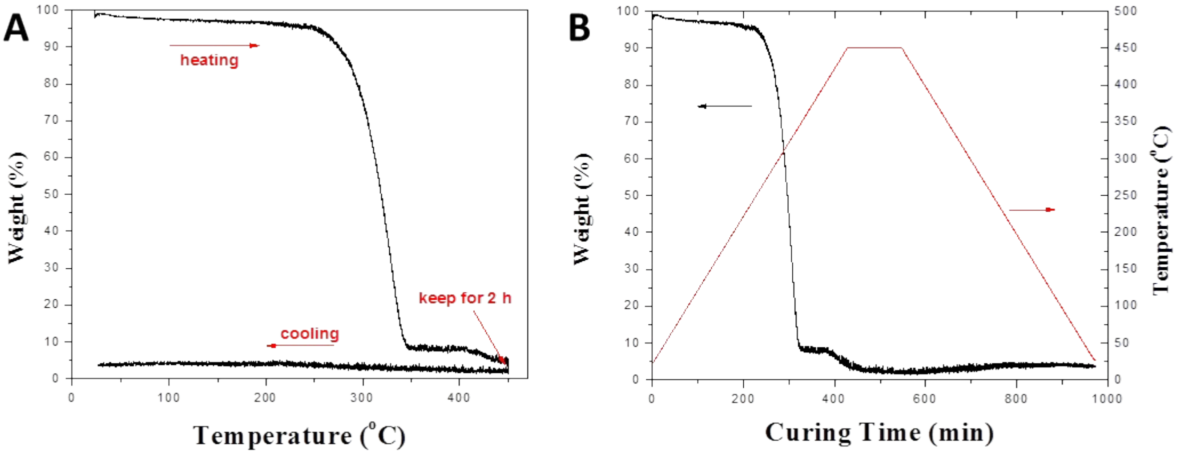

Figure S1. Thermal degradation of polystyrene sphere. TGAs showing 1000 nm PS particles that are used in making the porous materials have the typical degradation temperature at 240 °C. They will be fully degraded and be removed at 500 °C for a certain amount of time.

3. Critical Weight Ratio of Clay to PS for Open Pores Crack Free Film

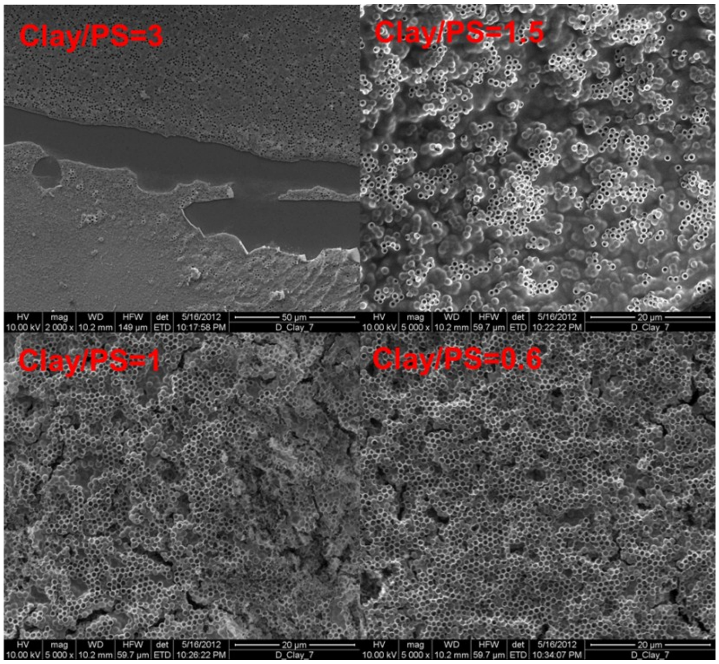

Figure S2. Open Pores Crack Free Film. Above the critical ratio of laponite to polystyrene (≥0.15), a crack free film was developed. The pores start to be closed and not to be interconnected at the ratio of 1. Further increase in ratio leads to embedded the pores inside the stacked laponite nanoplate layers. The size of polystyrene sphere is selected as ~1000 nm.

4. Cracking Properties of Inorganic Nanoplate Inverse Opal Structure

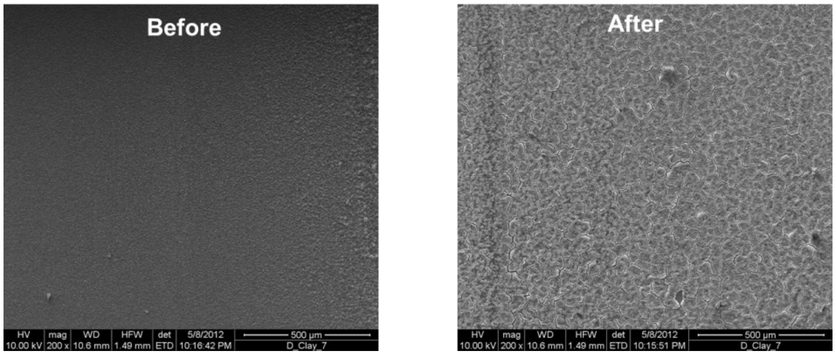

Figure S3. Mechanical Robustness of Laponite Nanoplate inverse opal film. After bending the substrate, a homogenously distributed set of cracks are formed.

Reference

1. O. Kammona, E. Dini, C. Kiparissides and R. Allabashi, *Microporous and Mesoporous Materials*, 2008, **110**, 141-149.
